# Early peanut introduction wins over the HLA-DQA1*01:02 allele in the interplay between environment and genetics

**DOI:** 10.1172/JCI155609

**Published:** 2022-01-04

**Authors:** Monali Manohar, Kari Christine Nadeau, Maya Kasowski

**Affiliations:** Sean N. Parker Center for Allergy and Asthma Research, Department of Medicine, Stanford University School of Medicine, Stanford, California, USA.

## Abstract

The rising incidence of food allergy in children underscores the importance of environmental exposures; however, genetic factors play a major role. How the environment and genetics interact to cause food allergy remains unclear. The landmark Learning Early About Peanut Allergy (LEAP) clinical trial established that early peanut introduction protects high-risk infants, consistent with the tolerizing effects of gut exposure. In this issue of the *JCI*, Kanchan et al. leveraged the LEAP trial data to examine molecular genetic mechanisms of early sensitization. A previously identified HLA risk allele for peanut allergy (DQA1*01:02) was associated with peanut-specific IgG4 levels in consumers. Notably, IgG4 antibodies likely provide protection by reducing the binding of allergen to IgE. The association of the same allele with peanut allergy in avoiders while potentially conferring protection in consumers reinforces the need to integrate genetic information toward a personalized therapeutic strategy for the best outcome in addressing food allergies.

## Contextual associations between HLA alleles and peanut allergy

The genetic factors that predispose an individual to atopy and food allergy including peanut have been evaluated through genome-wide association studies (GWAS) and candidate gene association studies (CGAS). Both approaches reveal a robust association between food allergy and HLA class II region (1), although how the interplay of genetic factors and environmental exposure, including early peanut introduction, modifies disease status had not been previously studied. In this issue of the *JCI*, Kanchan et al. (2) address this key gap through integrative analysis of multimodal data collected during the Learning Early About Peanut Allergy (LEAP) trial, a controlled peanut exposure study (3).

**Figure 1:**
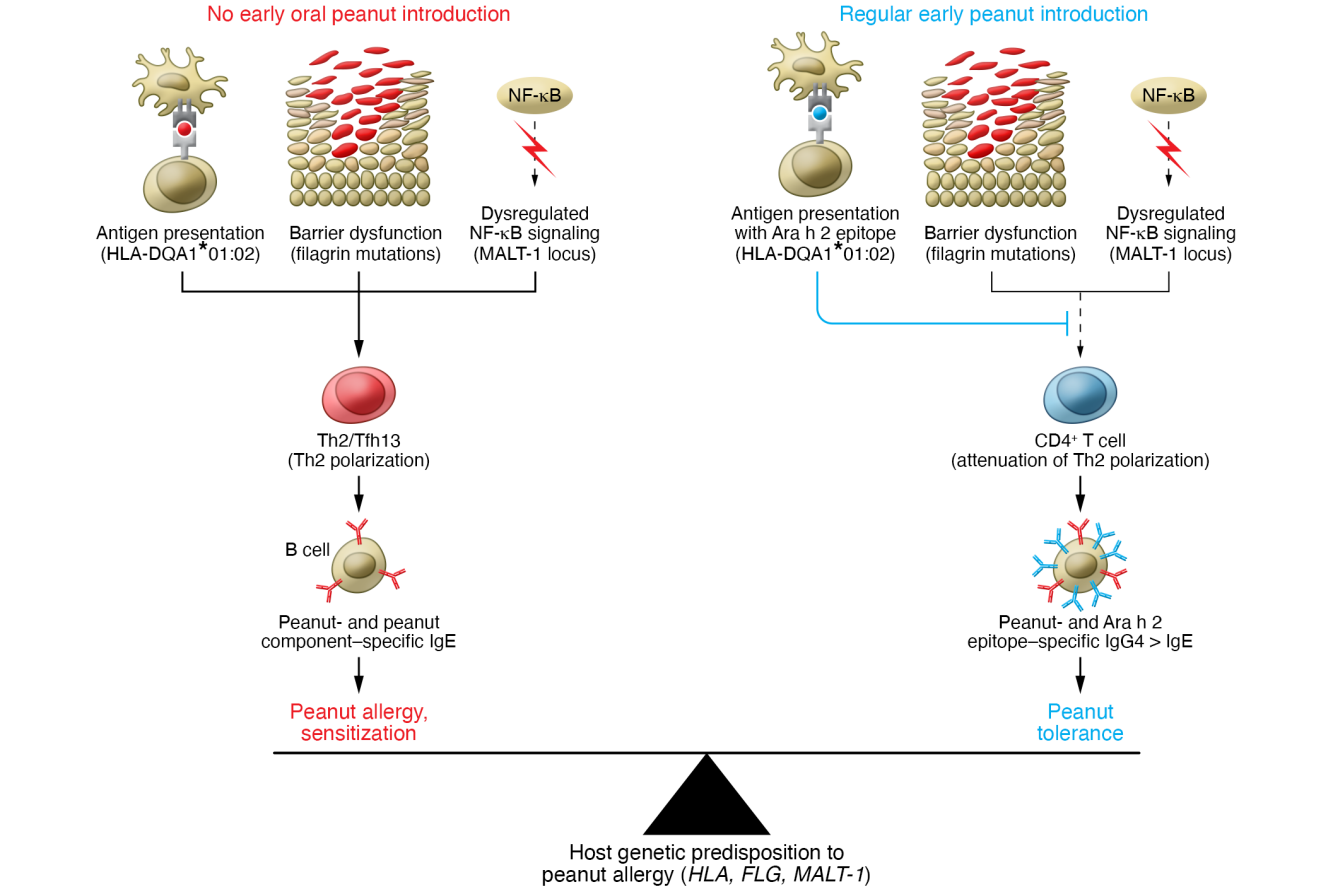
Tipping the balance in favor of peanut tolerance in the face of risk alleles. Genetic alleles, such as HLA-DQA*01:02, MALT-1, and FLG, predispose the carrier to the risk of developing peanut allergy. In the absence of oral peanut introduction in early infanthood (left panel), antigen presentation by HLA-DQA*01:02, barrier dysfunction caused by LFG mutations, and dysregulated NF-κB signaling due to MALT1 SNP together may promote polarization of naive CD4^+^ T cells to Th2 and Tfh13 cells. Th2 and Thfh13 secrete type 2 cytokines, including IL-4, IL-5, and IL-13, which induce IgE class switching in B cells. Peanut and peanut component–specific IgE thus generated leads to peanut allergy pathogenesis. With early oral peanut introduction (right panel), peanut-derived epitopes, specifically Ara h 2 epitopes presented in the context of HLA-DQA*01:02, likely counter Th2 polarization of naive CD4^+^ T cells. Attenuation of Th2 phenotype favors peanut epitope–specific IgG4 production over IgE production and could dampen the atopy-provoking effects of other risk alleles, thus leading to peanut tolerance.

Kanchan et al. (2) discovered an association between peanut-specific Ara h 2–specific IgG4 and the HLA-DQA1*01:02 allele among participants in the consumption arm, who were exposed to sustained oral peanut protein. This association was further validated through the observed sequential-epitope–specific IgG4 expansion in HLA-DQA1*01:02 carriers, but not noncarriers. Higher allergen-specific IgG4 has been linked with natural tolerance to a given allergen and is a biomarker of sustained unresponsiveness induced through allergen-specific immunotherapy (4, 5). Interestingly, the very same allele — HLA-DQA1*01:02 — was associated with peanut allergy risk in the avoidance arm of the study. These findings indicate that peanut epitope presentation to T helper cells by HLA-DQ may shape the host immune response to favor peanut tolerance versus allergy depending on modifiable environmental factors such as early peanut introduction (Figure 1).

## Roadmap for possible future HLA-related investigations

Future work may focus on more comprehensive genotyping of the HLA locus. It is important to consider, though, that all the HLA alleles in the host present various epitopes in concert, and studying the full complement of HLA allelic variants would best determine the cumulative impact of such presentation. Alternative pipelines affording parallel, comprehensive analysis of HLA-DR, -DP, and -DQ alleles are needed to effectively attribute immune response to the HLA molecule.

Kanchan and authors did not observe an association between HLA alleles and circulating (plasma) IL-4 and IL-10 levels in the consumption-arm participants (2). However, such evaluation would be more relevant had IL-4, IL-9, and IL-10 been induced through ex vivo peanut stimulation of participants’ PBMCs, thus representing cytokines secreted by peanut-reactive immune cells rather than measuring circulating levels.

Kanchan et al. invoked a dual–allergen exposure hypothesis, which posed that primary allergen exposure through skin is sensitizing and through the gut is tolerizing (2, 6). Thus, it would be worth comparing Ara h 2 epitope presentation via skin-resident and gastrointestinal-tract–resident antigen-presenting cells from HLA-DQA1*01:02 carriers using ex vivo assays.

## MALT1 is a risk allele with peanut epitope–independent effects

This research team previously associated the MALT1 SNP rs57265082, a paracaspase-encoding gene, with peanut allergy in the avoidance arm of LEAP. They also found *MALT1* to strongly associate with peanut-specific IgE as well as Ara h 1–, 2–, and 3-specific IgE, primarily in the avoidance group (7). In Kanchan et al. (2), the researchers observed *MALT1* to have no primary effect on peanut-, Ara h 2–, or other peanut component–specific IgG4. Also, the association of *MALT1* with peanut-specific IgE could not be attributed to certain sequential-epitope–specific expansion. Thus, the role of *MALT1* allele in peanut allergy pathogenesis is likely independent of allergen-epitope presentation. The paracaspase encoded by *MALT1* is a critical part of the CARMA1/BCL10/MALT1 complex, responsible for effective downstream activation of NF-κB in B and T cells in response to receptor-mediated stimulation. Hence, an intuitive mechanistic role of SNP rs57265082 MALT1 is to promote IgE class switching on a broader scale through dysregulated NF-κB signaling. Nevertheless, deeper investigation for better understanding the role of MALT1 signaling in peanut allergy is warranted. Also, MALT1 has not been identified as a risk allele in peanut allergy in general population GWAS (7). Thus, cross-validation studies in an independent cohort are needed.

The outcome for participants who carry risk alleles, including *FLG, DQ*, and *MALT1*, in the consumption arm after 12 months of avoidance (i.e., at week 72) in the context of LEAP-ON trial (8) is of interest too.

The results of Kanchan et al. (2) demonstrate the subtle, context-dependent effects of genetic risk factors. The alternate roles of HLA-DQA1*01:02 in the risk for peanut allergy and a favorable molecular response (Ara h 2 IgG4 levels) converge on a common mechanism of enhanced immune recognition. Whereas *MALT1* associates with the immune response to many peanut components, HLA-DQA1*01:02 is specific for Ara h 2, consistent with a narrower role (Figure 1). An important direction for future work involves exploring the generalizability of the LEAP story to other allergens. The dual-allergen exposure hypothesis is thought to apply broadly to food allergies. Are there other HLA allele–antigen dyads that convey risk in the absence of early consumption? Such data provide an understanding of the fundamentals of allergic sensitization and may also have practical clinical value for triaging at-risk individuals for oral immunotherapy (OIT) response that would confer lifelong benefit.

## Importance of GWAS in the context of therapeutic interventions

Kanchan et al. (2) also highlight the value of studying genetic effects in clinical trial cohorts. Although the scale is modest compared with that of GWAS, the LEAP trial continues to provide complementary and unique insights into the genetics of peanut allergy. A generalizable lesson from the LEAP experience may include the value of studying gene-environment interactions during critical developmental windows of exposure. Although HLA-DQA1*01:02 was previously identified by GWAS as a risk locus for peanut allergy, the link to peanut-specific IgG4 was uncovered by studying the response of high-risk infants to a potential therapeutic intervention. The clinical trial setting not only enabled the controlled perturbation of specific study populations, but also involved rich longitudinal phenotyping, including disease-relevant molecular studies (e.g., allergen-specific immunoglobulin levels). Enrolling children in clinical trials was essential to studying early etiologic events. For future investigations, it will also be important to recruit individuals of non-European–derived genetic ancestries in order to understand population differences in allergy, prioritize causal variants, and address iniquities in the allocation of GWAS resources. One can imagine replicating the success of the LEAP trial to study the impact of other developmentally timed exposures that may moderate the effects of genetic susceptibility (e.g., microbial exposures). Studying high-risk children has the benefit of revealing therapeutic strategies for those in greatest need. In addition, the large effect sizes (as observed in the LEAP cohort) may also help identify informative contexts for large-scale, general population analyses.

As the authors point out, consumption is so effective in preventing peanut allergy that it is not possible from their data to assess whether HLA-DQA1*01:02 associates with tolerance in consumers. Thus, the clinical relevance of this gene-environment interaction awaits future work. Despite the limitations, Kanchan et al. (2) undoubtedly highlight how a therapeutic intervention such as peanut consumption through OIT may differentially affect participants with varying genotypes. Although it remains to be tested, past and current LEAP analyses raise the intriguing possibility that early childhood genotyping may help prioritize at-risk individuals for OIT.

## Version 1 01/04/2022

Electronic publication
